# Digital expression profile of immune checkpoint genes in medulloblastomas identifies CD24 and CD276 as putative immunotherapy targets

**DOI:** 10.3389/fimmu.2023.1062856

**Published:** 2023-02-07

**Authors:** Rui Ferreira Marques, Daniel Antunes Moreno, Luciane da Silva, Leticia Ferro Leal, Flávia Escremim de Paula, Iara Santana, Gustavo Teixeira, Fabiano Saggioro, Luciano Neder, Carlos Almeida Junior, Bruna Mançano, Rui Manuel Reis

**Affiliations:** ^1^ Life and Health Sciences Research Institute (ICVS), School of Health Sciences, University of Minho, Braga, Portugal; ^2^ ICVS/3B’s –PT Government Associate Laboratory, Braga, Guimarães, Portugal; ^3^ Molecular Oncology Research Center, Barretos Cancer Hospital, Barretos, Brazil; ^4^ Faculty of Health Sciences of Barretos Dr. Paulo Prata (FACISB), School of Medicine, Barretos, Brazil; ^5^ Laboratory of Molecular Diagnostic, Barretos Cancer Hospital, Barretos, Brazil; ^6^ Department of Pathology, Barretos Cancer Hospital, Barretos, Brazil; ^7^ Department of Pathology and Forensic Medicine, Ribeirão Preto Medical School, University of São Paulo, Ribeirão Preto, Brazil; ^8^ Barretos Children’s Cancer Hospital, Barretos, Brazil

**Keywords:** medulloblastoma, immune checkpoints, immunotherapy, nCounter mRNA expression assay, immune profile

## Abstract

**Introduction:**

Medulloblastoma is the most common and lethal pediatric malignant brain tumor. It comprises four main molecular subgroups: WNT-activated, SHH-activated, Group 3, and Group 4. Medulloblastoma treatment is surgical resection, craniospinal radiation, and chemotherapy. However, many patients do not respond to therapy, and most suffer severe side effects. Cancer immunotherapy targeting immune checkpoints (IC) (PD-1, PD-L1, and CTLA4) has been getting disappointing outcomes in brain tumors. Nevertheless, other less explored immune checkpoints may be promising candidates for medulloblastoma therapy.

**Objectives:**

In the present study, we aimed to characterize the expression profile of 19 immune checkpoints in medulloblastoma.

**Methods:**

We analyzed 88 formalin-fixed paraffin-embedded medulloblastomas previously classified for each molecular subgroup and three non-tumoral brain tissue. mRNA levels of 19 immune checkpoint-related genes were quantified using the nCounter (PanCancer Immune Profiling Panel) assay. Further *in silico* analysis was performed in two larger public microarray datasets, one of which enabled comparisons between tumoral and non-tumoral tissues. Immunohistochemistry of PD-L1 was performed in a subset of cases. Microsatellite instability was also molecularly analyzed.

**Results:**

We observed an absence of expression of the canonic ICs, namely *PDCD1* (PD-1), *CD274* (PD-L1), and *CTLA4*, as well as *CD80, CD86, BTLA, IDO1, CD48, TNFSF14, CD160, CEACAM1*, and *CD244.* PD-L1 protein expression was also practically absent. We found higher mRNA levels of *CD24, CD47*, *CD276* (B7-H3), and *PVR*, and lower mRNA levels of *HAVCR2*, *LAG3*, and *TIGIT* genes, with significant differences across the four molecular subgroups. Compared to the non-tumor tissues, the expression levels of *CD276* in all subgroups and *CD24* in SHH, Group 3, and Group 4 subgroups are significantly higher. The *in silico* analysis confirmed the expression profile found in the Brazilian cohort, including the lower/absent expression of the canonic ICs. Moreover, it confirmed the overexpression of *CD24* and *CD276* in medulloblastomas compared with the non-tumor tissue. Additionally, *CD276* and CD24 high levels were associated with worse survival.

**Conclusion:**

These results highlight the low or absence of mRNA levels of the canonic targetable ICs in medulloblastomas. Importantly, the analysis revealed overexpression of *CD24* and *CD276*, which can constitute prognostic biomarkers and attractive immunotherapy targets for medulloblastomas.

## Introduction

Central nervous system (CNS) tumors are children’s and teenagers’ second most common malignancies. Medulloblastoma is the most prevalent and deadliest in pediatric patients (1). Medulloblastoma is histologically classified into five groups: classic, desmoplastic/nodular, extensive nodularity, large cell, and anaplastic (2). The latest World Health Organization (WHO) classification further divides medulloblastoma into four molecularly defined subgroups: WNT-activated, SHH-activated *TP53* wildtype, SHH-activated *TP53* mutated, and the non-WNT/non-SHH, which comprises two different subgroups numerically named “3” (G3) and “4” (G4) (3). These molecular subgroups are associated with particular genetic and clinical features, leading to specific prognostication. (1, 3–5).

The survival of medulloblastoma patients has increased in the last decade, mainly due to improvements in treatment strategies such as maximum safe surgical resection, craniospinal radiation in patients older than three years, and chemotherapy. However, the survival for high-risk patients remains low; many patients do not respond to therapy, and most suffer from severe side effects, mainly because of radiation effects on the brain (1, 6). Therefore, there is an urgent need to develop new, more effective, and less toxic therapies (6).

Cancer immunotherapy using immune checkpoint (IC) blockade has shown promising results in treating solid tumors (7, 8). Currently, PD-1, PD-L1, and CTLA4 are the best-established targets, already having FDA-approved monoclonal antibodies for the treatment of many different cancer types (9; 10). However, applying a single immune checkpoint blocker for these targets in CNS tumors, particularly medulloblastoma, did not attain the expected results in clinical trials, particularly in pediatric patients (11–13). Clinical trials using PD-1 inhibitors such as pembrolizumab and nivolumab have shown minor clinical significance in patients with CNS tumors, including in medulloblastoma (11–13).

Alternatively to the currently actionable checkpoints mentioned, other immune checkpoints could become attractive targets of blockage, such as B7-H3, Tim-3, IDO, CD47, LAG-3, TIGIT, and PVR (14–16). Additionally, the knowledge of the non-canonic checkpoints is still poorly described in medulloblastoma and could represent new promising targets for therapy (9, 17). Hence, the immune checkpoint expression profile characterization in medulloblastomas is of utmost importance and could provide crucial cues for more effective and appropriate immune checkpoint blockade-based immunotherapy (10, 17).

The present study aimed to characterize the immune checkpoint profile in medulloblastomas, assess whether there is a distinct profile among molecular subgroups, and evaluate its prognostic outcome.

## Materials and methods

### Study population

We included 88 formalin-fixed paraffin-embedded (FFPE) medulloblastoma specimens from patients diagnosed at Barretos Cancer Hospital (BCH), Barretos, and Ribeirão Preto Medical School, Brazil, from 1986 to 2018. Experienced pathologists reviewed the histology, and the tumors were previously molecularly characterized by a 22-gene nCounter assay into WNT (n=14), SHH (n=43), Group 3 (n=12), and Group 4 (n=19) (5, 18, 19). This study was approved by the Ethics Committee in Research from Barretos’ Cancer Hospital (Project #1248/2016). Additionally, three non-tumoral samples derived from non-tumor tissue surrounding brain tumor metastasis were included in the study.

### DNA and RNA isolation

Following tumor area demarcation, ensuring the presence of >60% of tumor content by an experienced pathologist, DNA and RNA were isolated from FFPE samples (20). DNA was isolated using the QIAamp DNA Mini Kit (Qiagen, Venlo, The Netherlands) and quantified using NanoDro*PVR* 2000 (Thermo Scientific, Waltham). RNA isolation and quantification were performed using the RNeasy Mini Kit (Qiagen, Venlo, The Netherlands) and NanoDro*PVR* 2000 (Thermo Scientific, Waltham).

### Microsatellite instability status

Microsatellite instability analysis was performed using the Human Target – Microsatellite Instability Plus (HT-MSI+) from Cellco (São Carlos, Brazil), which consists of a multiplex PCR with six quasi-monomorphic mononucleotide repeat markers: BAT-25, BAT-26, NR- 21, NR-24, NR-27 and HSP110 (21). The assay was performed using 0.5 µL of DNA at 50 ng/mL and reverse primers end-labeled with fluorescent dyes. Allele size with a range of plus or minus three nucleotides established each marker’s quasimonomorphic variation range (QMVR) (22, 23).

### Immune checkpoint gene expression analysis

Gene expression assays were performed in the nCounter^®^ FLEX Analysis System, using the PanCancer Immune Profiling Panel (NanoString Technologies, Inc., Seattle, WA). This panel comprises 730 immuno-oncology-related targets, including 109 cell surface markers for 14 immune cell types and 40 reference genes (https://nanostring.com/products/ncounter-assays-panels/oncology/pancancer-immune-profiling/) (24).

For downstream analysis, we selected 19 immune checkpoints related genes described in the literature, including: *PDCD1* (PD-1), *CD274* (PD-L1), *CTLA4*, *CD276* (B7-H3), *LAG3*, *PVR* (CD155), *CD47*, *CD80*, *CD86*, *BTLA*, *IDO1*, *HAVCR2* (TIM-3), *CD48*, *TNFSF14*, *CD160*, *CEACAM1*, *CD244*, *TIGIT* and *CD24*. (25–32).

Quality control parameters such as binding density, detection limit, positive controls, and housekeeping counts were measured using the nSolver™ Analysis Software v4.0 (NanoString Technologies) and ROSALIND^®^. Samples presenting less than 30% of housekeeping genes above 50 counts were removed from the analysis. Additionally, raw data were normalized using housekeeping genes through the ROSALIND^®^ platform (33), and absence was considered when counts were below 20, the background threshold level. The normalized data was employed as input for downstream analysis, and the statistical analysis for subgroups’ mRNA level comparisons was performed using the IBM SPSS version 27.

### *In silico* analysis

Immune checkpoints’ mRNA levels were validated using two datasets downloaded from ‘R2: Genomics Analysis and Visualization Platform (http://r2.amc.nl )’. The Cavalli et al. microarray dataset (Affymetrix Gene 1.1 ST array, GSE85218) consists of 763 medulloblastomas divided into the four molecular subgroups: WNT (n=70), SHH (n=223), Group 3 (n=144) and Group 4 (n=326), containing also overall survival data (34). The second dataset consists of the fusion of several different datasets of medulloblastoma microarray analysis normalized through RUV (remove unwanted variation). This dataset, from now on called the Batch dataset, comprises 291 normal brain samples and 1350 medulloblastomas also divided into the four molecular subgroups: WNT (n=118), SHH (n=405), Group 3 (n=233) and Group 4 (n=530) (GSE124814).

### PD-L1 immunohistochemistry

Immunohistochemistry of PD-L1 in 29 medulloblastoma samples was performed using the Dako EnVision FLEX + HRP-polymer kit (22C3 clone). The slides were submitted to deparaffinization and rehydration, followed by antigen retrieval (Dako EnVision FLEX Target Retrieval pH6). Staining was performed in the Dako Automated Link 48, and the PD-L1 antibody was prepared following the manufacturer’s instructions and previous studies, using the lung cancer cell line NCI-H226 as a positive control (35). Lastly, the slides were counterstained with hematoxylin. The intensity staining was determined as follows: 1+ (weak), 2+ (moderate) or 3+ (strong) (36).

### Statistical analysis

Comparisons between immune checkpoints mRNA levels in medulloblastoma molecular subgroups were made using a parametric approach. The homogeneity of variances was verified using Levene’s test. Accordingly, when homogeneity of variances was observed, OneWay-ANOVA was applied using the Tukey HSD method for multiple comparisons. When no homogeneity of variances was observed the Welch test for robust comparisons was applied, using the Games-Howell method for multiple comparisons. Differentially expressed genes were considered when the adjusted p-value was lower than 0.05. The Cavalli scaled data was obtained through the “scale” function of R software and the heatmap made in GraphPad 8.

The evaluation of overall survival was performed by the Kaplan-Meier method, with the p-value established by log-rank and Gehan-Breslow-Wilcoxon tests using the GraphPad Prism 8 software. The median of digital counts was established as a cutoff point for the stratification into high/low mRNA levels. It was also defined the hazard ratio for each gene.

## Results

Eight of the 88 medulloblastomas evaluated by nCounter were excluded due to low housekeeping counts, leading to a final number of 80 high-quality samples for the gene expression analysis. The cases were molecularly divided into WNT (n=13, 16.2%), SHH (n=39, 48.8%), Group 3 (n=10, 12.5%), and Group 4 (n=18, 22.5%). The major clinicopathological features are summarized in **Table 1**.

**Table 1:** Clinicopathological features of the 80 Brazilian medulloblastomas.

Variables		WNT (n=13)	SHH (n=39)	Group 3 (n=10)	Group 4 (n=18)
		N (%)	N (%)	N (%)	N (%)
**Gender**	Male	5 (38.5%)	25 (64.1%)	7 (70%)	12 (66.7%)
	Female	8 (61.5%)	14 (35.9%)	3 (30%)	6 (33.3%)
**Age group**	Infant (< 3)	0 (0%)	3 (7.7%)	2 (20%)	1 (5.6%)
	Child (> 3 - 12)	6 (46.2%)	4 (10.3%)	6 (60%)	12 (66.7%)
	Teenager (> 12 - 18)	3 (23.1%)	4 (10.3%)	2 (20%)	2 (11.1%)
	Adult (> 18)	4 (30.8%)	28 (71.8%)	0 (0%)	3 (16.7%)
**Histological subtype**	Classic	10 (76.9%)	12 (30.8%)	7 (70%)	16 (88.9%)
	Extensive nodularity	2 (15.4%)	2 (5.1%)	1 (10%)	1 (5.6%)
	Nodular/Desmoplastic	0 (0%)	19 (48.7%)	1 (10%)	0 (0%)
	Anaplastic/Large cells	1 (7.6%)	1 (2.6%)	1 (10%)	0 (0%)
	Missing	0 (0%)	5 (12.8%)	0 (0%)	1 (5.6%)
**Metastasis at diagnostics**	Yes	0 (0%)	4 (10.3%)	3 (30%)	12 (66.7%)
	No	13 (100%)	34 (87.2%)	7 (70%)	6 (33.3%)
	Missing	0 (0%)	1 (2.6%)	0 (0%)	0 (0%)
**Surgical resection**	Total	6 (46.1%)	21 (53.8%)	5 (50%)	9 (50%)
	Partial	7 (53.8%)	18 (46.2%)	5 (50%)	9 (50%)
	Missing	0 (0%)	1 (2.6%)	0 (0%)	0 (0%)
**Status**	Alive	8 (61.5%)	18 (46.2%)	6 (60%)	11 (61.1%)
	Dead	4 (30.8%)	20 (51.3%)	4 (40%)	7 (38.8%)
	Loss of follow up	1 (7.7%)	1 (9.1%)	0 (0%)	0 (0%)

“n”, sample size; “N”, number of patients.

The MSI status was successfully analyzed in 56 (70%) cases, and all of them were classified as microsatellite stable (MSS).

### Low/absence of mRNA levels of canonic actionable immune checkpoints *PDCD1 (*PD-1)*, CD274* (PD-L1)*, and CTLA4* in medulloblastomas

**Figure 1:**
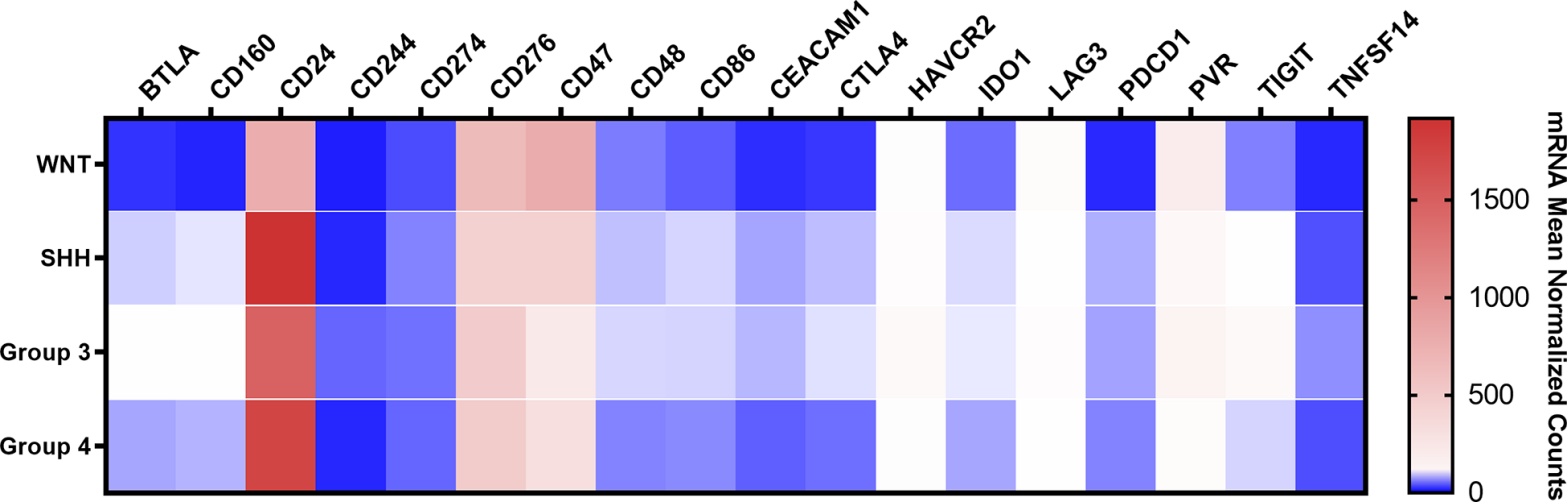
Heatmap of the mRNA levels of 19 immune checkpoint-related genes in the Brazilian 80 medulloblastomas. The color scale represents the mean normalized mRNA counts of the evaluated genes. Blue represents counts below the background threshold; white represents low counts (below 100 counts); red represents higher counts. The graph was obtained through GraphPad Prism 8.

**Figure 2:**
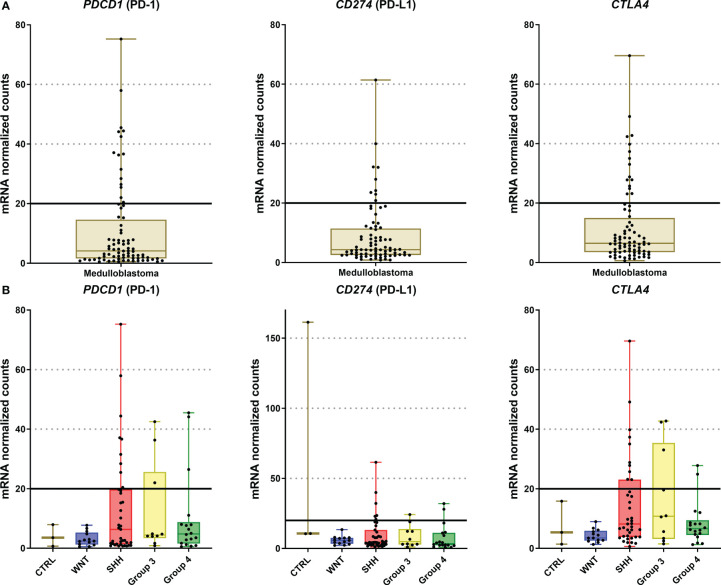
Graphic representation of PDCD1, CD274, and CTLA4 mRNA levels of the 80 medulloblastomas analyzed by nCounter. **(A)** The plot of mRNA levels of all medulloblastomas for PDCD1, CD274, and CTLA4; **(B)** Plot of PDCD1, CD274, and CTLA4 mRNA levels by medulloblastoma molecular subgroups. The continuous line in each graph marks the background threshold of 20 mRNA normalized expression counts. The plots were obtained through GraphPad Prism 8.

**Figure 3:**
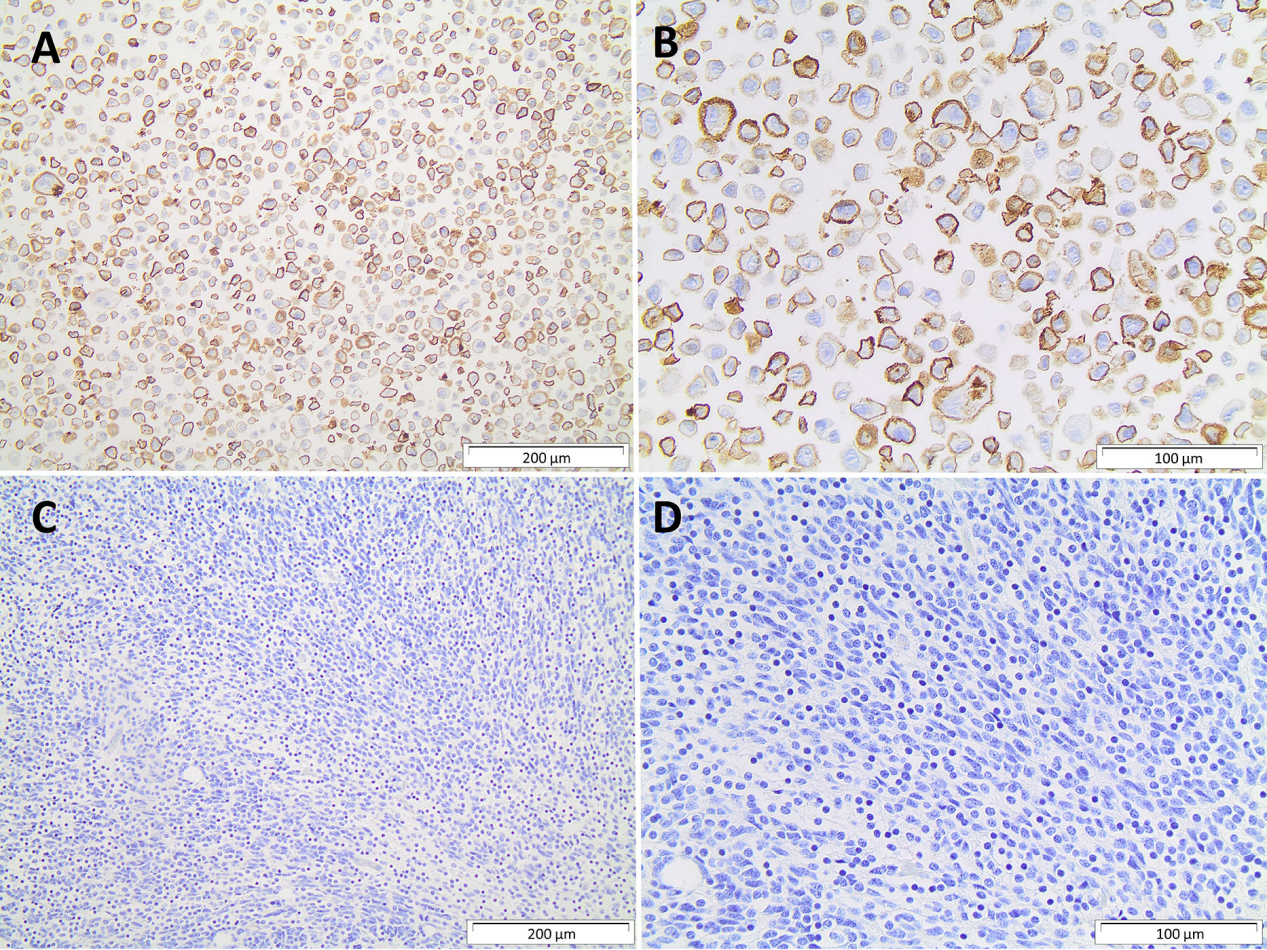
Immunohistochemistry PD-L1 protein. **(A, B)** Strong membrane immunostaining of positive control with 200µm and 400µm magnification, respectively; **(C, D)** absence of PD-L1 immunostaining in a medulloblastoma, 200µm and 400µm magnification, respectively.

The immune checkpoint analysis of the 19 genes in our series of 80 medulloblastomas showed overall low mRNA levels, particularly of the canonic actionable targets *PDCD1 (*PD-1*)*, *CD274* (PD-L1), and *CTLA4* (**Figure 1**). The mRNA normalized counts for *PDCD1* (PD-1) ranged from 0.5 to 75.8, with a mean value of 10.7, being below the background threshold level of 20 counts and therefore considered an absence of expression (**Figure 2A**). Likewise, *CD274* (PD-L1) mean mRNA levels were below the background threshold. The absence of *CD274* expression was also observed by the lack of PD-L1 immunohistochemistry staining in the 29 cases evaluated (**Figures 3A–D**). Similarly, *CTLA4* mRNA levels were also below the threshold (mean counts = 11.7). The overall expression values of all genes are shown in **Table 2** and **Supplementary Table 1**.

**Table 2:** Mean mRNA normalized counts of the immune checkpoints evaluated in the Brazilian and Cavalli cohorts.

Genes	Medulloblastoma		WNT		SHH		Group 3		Group 4	
	Brazil (n=80)	Cavalli dataset (n=763)	Brazil (n=13)	Cavalli dataset (n=70)	Brazil (n=39)	Cavalli dataset (n=223)	Brazil (n=10)	Cavalli dataset (n=144)	Brazil (n=18)	Cavalli dataset (n=326)
***BTLA***	13,6	15,9	3,9	15,4	15,9	15,8	20,6	16,8	12,8	15,6
***CD160***	15,3	9,3	2,8	9,1	17,7	9,4	28,3	9,6	13,8	9,1
***CD24***	1562,4	1069,4	763,7	554,2	1889,1	1086,9	1459,4	823,4	1710,4	1276,7
***CD244***	3,5	12,0	2,3	12,1	2,9	12,1	7,9	12,5	2,9	11,8
***CD274***	10,6	15,1	5,8	21,3	10,1	16,3	8,7	14,7	7,8	13,1
***CD276***	472,1	346,6	633,4	424,6	432,0	297,0	476,2	315,0	487,3	377,8
***CD47***	442,8	840,8	772,6	1224,5	434,2	1011,6	211,7	646,2	305,9	727,5
***CD48***	13,4	30,2	9,5	32,8	14,8	32,7	16,5	27,0	10,1	29,3
***CD80***	10,9	15,8	3,5	17,6	13,7	15,7	13,1	17,0	9,2	15,1
***CD86***	14,4	31,1	7,1	30,0	16,4	36,4	16,3	27,1	10,6	29,5
***CEACAM1***	10,1	17,9	3,4	17,2	12,7	19,7	14,1	17,3	7,3	17,2
***CTLA4***	11,7	13,5	4,2	12,9	14,6	13,9	17,2	13,7	8,5	13,3
***HAVCR2***	44,1	52,1	33,0	52,3	43,0	62,9	66,7	44,9	33,7	47,9
***IDO1***	14,3	16,6	8,3	15,1	16,9	15,1	18,0	25,9	12,8	13,9
***LAG3***	27,9	61,8	47,7	69,9	21,1	57,0	40,7	67,0	23,6	61,1
***PDCD1***	10,7	47,8	3,2	45,3	13,5	46,4	12,5	46,8	10,1	49,9
***PVR***	101,4	134,0	187,4	252,0	86,2	150,8	124,9	163,6	50,2	84,0
***TIGIT***	23,6	12,6	9,9	12,7	20,8	13,2	68,9	12,2	16,3	12,3
***TNFSF14***	6,9	41,5	3,0	45,0	6,1	40,0	11,0	43,1	6,0	41,1

Grey genes mark the mean counts above the background threshold.

There were no significant differences between medulloblastoma molecular subgroups and non-tumor tissues in *PDCD1*, *CD274*, and *CTLA4* levels (**Figure 2B** and **Supplementary Table 3**).

**Figure 4:**
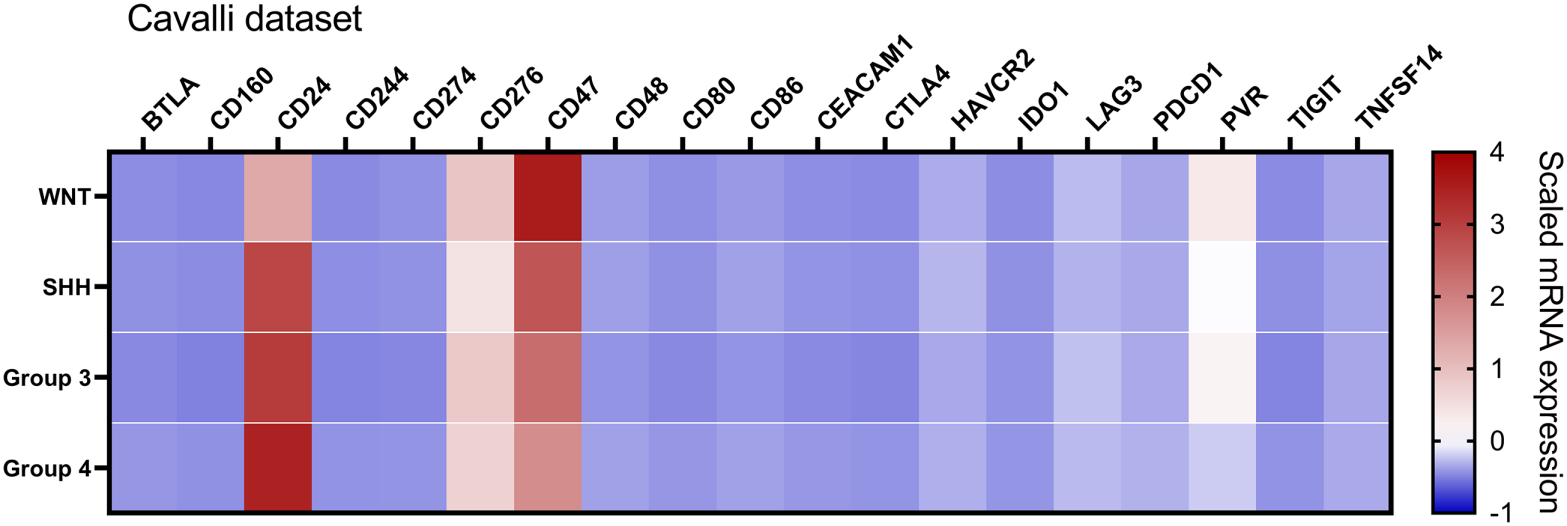
Heatmap of the mRNA levels of 19 immune checkpoint-related genes in the Cavalli et al. dataset. The color scale represents the scaled mRNA. The graph was obtained through GraphPad Prism 8.

To further extend and validate the findings observed in our Brazilian medulloblastoma series, we performed an *in silico* analysis of immune-checkpoints mRNA levels in the Cavalli et al. microarray dataset. We could corroborate in this large dataset of 763 medulloblastomas the low levels of *PDCD1 (PD-1)*, *CD274* (PD-L1), and *CTLA4* (**Figure 4**, **Table 2**, and **Supplementary table 2**).

### Higher mRNA expression levels of *CD24, CD276* (B7-H3)*, CD47, and PVR* immune checkpoints in medulloblastomas

**Figure 5:**
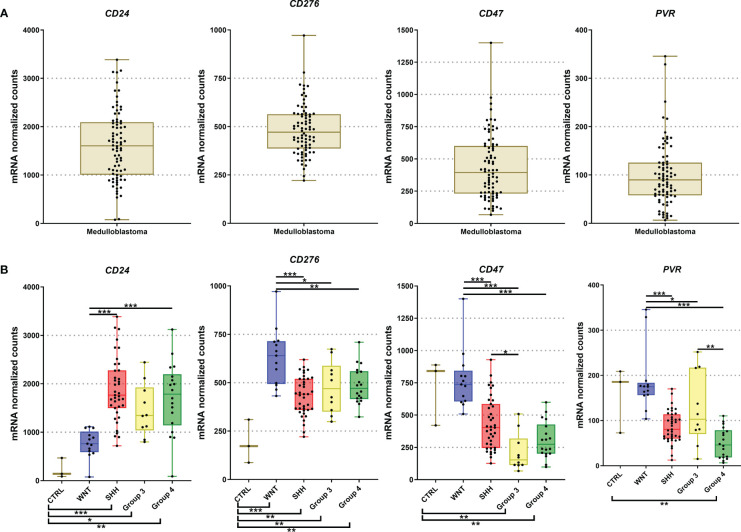
Graphic representation of CD24, CD276, CD47, and PVR mRNA levels obtained from our 80 medulloblastomas cohort submitted for analysis by nCounter assay. **(A)** The plot of mRNA levels of all medulloblastomas for CD24, CD276, CD47, and PVR; **(B)** Plot of CD24, CD276, CD47, and PVR mRNA levels by molecular subgroups. The significance level is 0.05. * < 0.05; * < 0.01; *** < 0.001. The plots were obtained through GraphPad Prism 8.

Among the 19 immune checkpoint-related genes evaluated by nCounter in the Brazilian series of medulloblastomas, we found increased mRNA counts of *CD24*, *CD276*, *CD47*, and *PVR* (CD155) (**Figure 1**). The highest expressed gene was *CD24* with mean normalized counts of 1562.4, followed by the *CD276* (mean counts = 472.1), *CD47* (mean counts = 442.8), and finally, the *PVR* gene (mean counts = 101.4) (**Figure 5A** and **Table 2**). *CD24* expression was high across all molecular subgroups when compared with non-tumor tissues, and it showed significantly higher levels in the SHH and Group 4 than in the WNT subgroup (**Figure 5B**). The second highest expressed gene was *CD276*, which exhibited a significantly higher expression in all subgroups compared to non-tumor tissues. The WNT subgroup had the highest CD276 expression (mean counts = 633.4) compared to SHH (mean counts = 432.0), Group 3 (mean counts = 476.2), Group 4 (mean counts = 487.3) subgroups (**Figure 5B**). The *CD47* gene displayed high expression levels across the four subgroups and the non-tumor tissue (**Figure 5B**). It showed significantly overexpressed in the WNT subgroup compared with SHH, Group 3, and Group 4 subgroups (**Figure 5B**). The SHH subgroup also showed significantly higher mRNA levels of *CD47* than Group 3. *CD47* expression in the non-tumor tissues was similar to the WNT subgroup and presented a significantly higher expression than in the subgroups 3 and 4 (**Figure 5B**). Lastly, *PVR* showed significantly higher mRNA levels in the WNT subgroup, followed by Group 3, SHH, and Group 4 (**Figure 5B**). Moreover, Group 4 showed significantly lower expression levels of *PVR* when compared to the non-tumoral samples (**Figure 5B**). All statistical comparisons are reported in **Supplementary Tables 3**, **4**.

**Figure 6:**
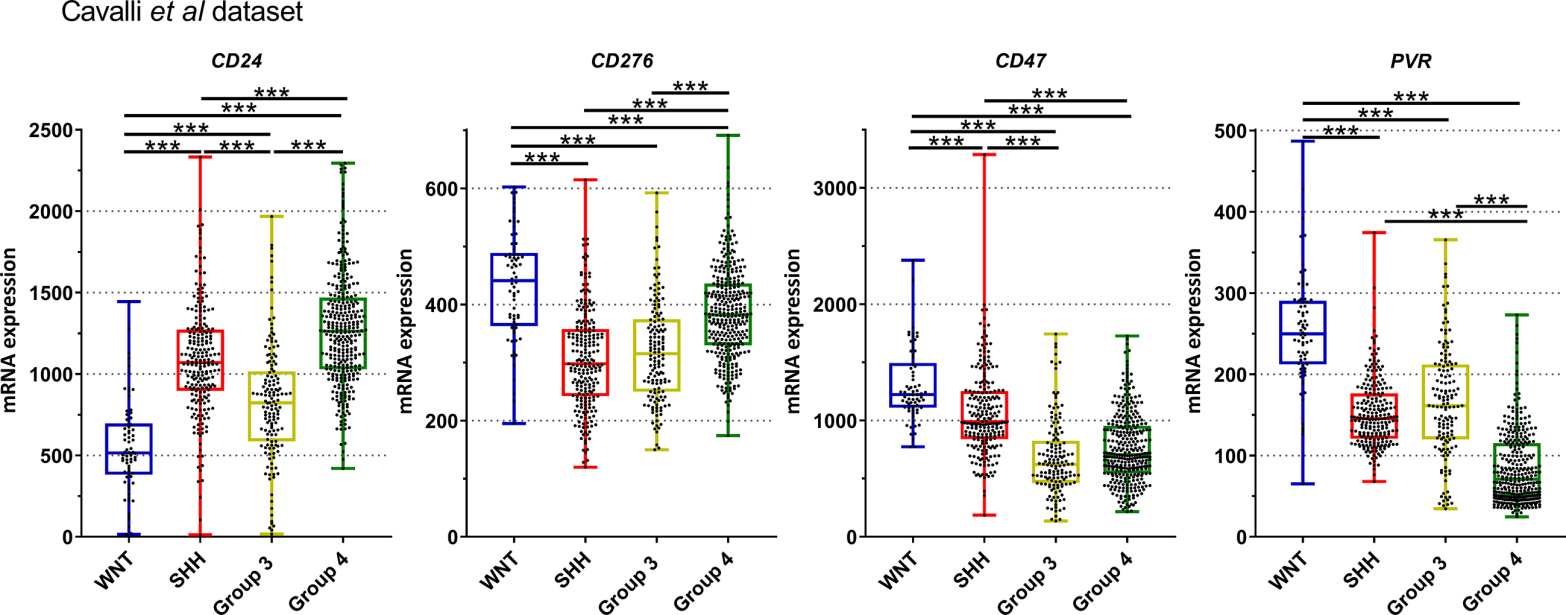
Graphic representation of *CD24*, *CD276*, *CD47*, and *PVR* mRNA normalized mRNA levels obtained from Cavalli et al. dataset. The significance level is 0.05. * < 0.05, ** < 0.01, *** < 0.001. The plots were obtained through GraphPad Prism 8.

Similar trends were observed in the Cavalli et al. microarray dataset, which showed high mRNA levels of *CD24*, *CD47*, *CD276*, and *PVR* genes (**Figures 4**, **6**). In this dataset, the highest expressed gene was *CD24* (mean count = 1069.4), followed by *CD47* (mean counts = 840.8), CD276 (mean counts = 346.6), and *PVR* (mean counts = 134.0) (**Figures 4**, **6**, and **Table 2**).

**Figure 7:**
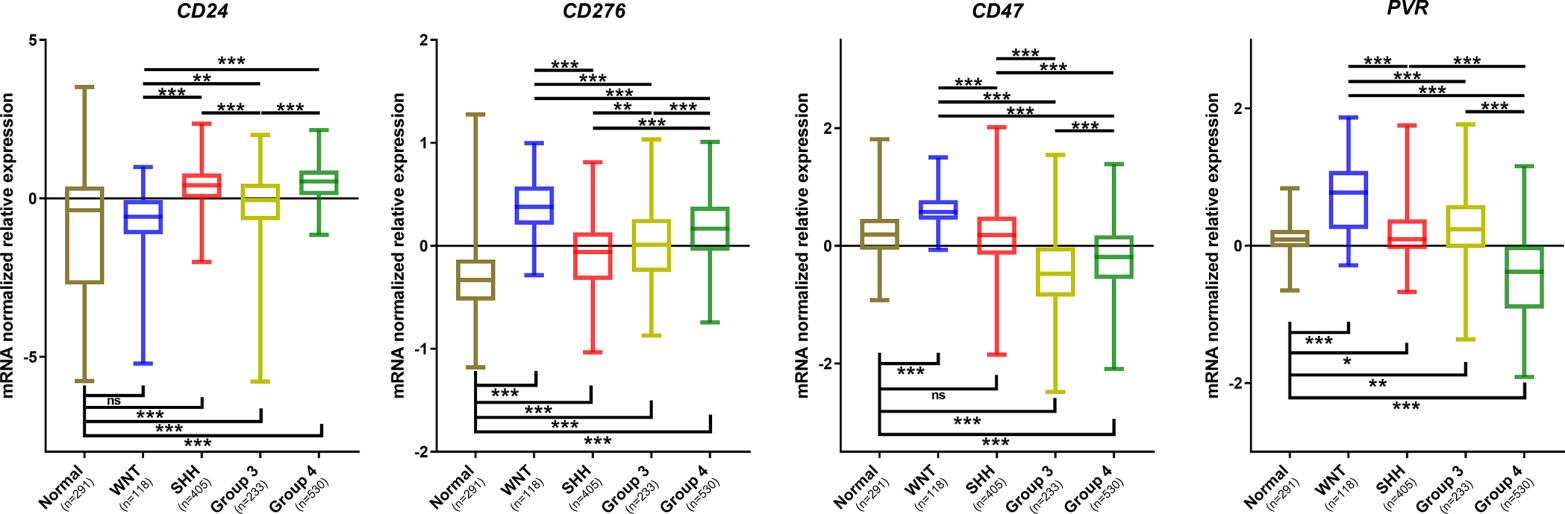
Graphic representation of *CD24*, *CD276*, *CD47*, and *PVR* mRNA normalized mRNA levels obtained from the analysis of the Batch dataset. The significance level is 0.05. * < 0.05; ** < 0.01; *** < 0.001. The plots were obtained through GraphPad Prism 8. ns, non significant.

Further *in silico* analysis was performed in an additional dataset comprised of 1350 medulloblastomas and 291 normal cerebellum cases (**Figure 7**). Similar to our Brazilian cohort, we found a similar expression profile of *CD24*, *CD47*, *CD276*, and *PVR* across the four subgroups and the non-tumoral samples (**Figure 7**). Regarding *CD24* levels, the SHH and Group 4 medulloblastomas showed higher expression levels and the WNT subgroup, and the non-tumor tissue presented significantly lower expression levels than the remaining subgroups (**Figure 7**). The expression profile of *CD47* was significantly higher in the WNT compared to the other subgroups and non-tumor tissue. Moreover, the non-tumor tissue also depicted higher expression levels than SHH, Group 3 and 4 (**Figure 7**). CD276 was overexpressed in all subgroups compared to the non-tumor tissues, like in our Brazilian dataset. Additionally, the WNT subgroup showed the higher expression, and the SHH subgroup presents with the lower expression than the other subgroups, followed by Group 3 and Group 4 subgroups. Lastly, *PVR* showed the highest expression in the WNT subgroup compared with the subgroups and non-tumor tissue. The non-tumor tissue showed higher levels than Group 4 and lower than SHH and Group 3 (**Figure 7**). The statistical values are reported in **Supplementary Tables 5**, **6**.

Moreover, in the Brazilian cohort, low mean mRNA levels were observed for *TIGIT* (mean counts = 23.6), *LAG3* (mean counts = 27.9), and *HAVCR2* (TIM-3) (mean counts = 44.1) genes (**Figure 1**, **Table 2** and **Supplementary Figure 1**). Finally, the analysis of the other immune checkpoints, *CD80, CD86, BTLA, IDO1, CD48, TNFSF14, CD160, CEACAM1*, and *CD244*, exhibited an absence of expression since the mean counts were below the established threshold levels (**Figure 1** and **Table 2**).

### Expression of *CD276* (B7-H3) and *CD24* are associated with worse patient outcomes

We further assessed the association of *CD276*, *CD47*, *CD24*, and *PVR* expression profiles by nCounter and patient survival analysis in the 80 medulloblastomas Brazilian cohort. No significant difference in overall survival was observed (**Supplementary table 7**).

**Figure 8:**
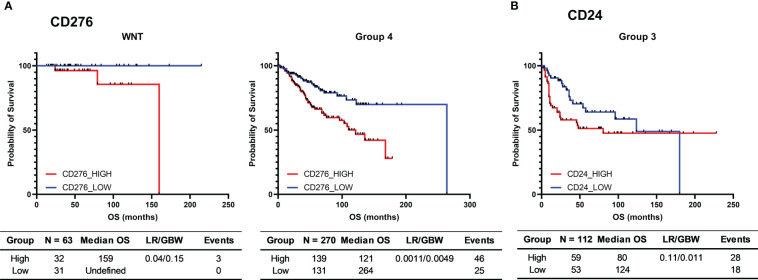
Kaplan-Meier curves for the survival of medulloblastoma patients from the 763 medulloblastomas Cavalli et al. cohort. **(A)** Probability of survival of *CD276* low and high WNT and Group 4 medulloblastoma patients; **(B)** Probability of survival of *CD24* low and high Group 3 medulloblastoma patients. High and low groups were established using the median as the cutoff point. The tables report the number of cases (N), median overall survival (Median OS), p-value obtained through the Log-rank test (p-value), hazard ratio and its associated 95% confidence interval, and the number of deaths (Events). Survival time is presented in months.

The same outcome analysis, performed *in silico* in the Cavalli et al. microarray dataset (**Supplementary Table 8**), showed that patients with high *CD276* mRNA levels presented a significantly shorter overall survival in the subgroups WNT (log-rank = 0.040) and Group 4 (log-rank = 0.0011 and GBW = 0.0049) (**Figure 8A**). Additionally, higher expression of *CD24* in Group 3 was associated with significantly worse survival only in the GBW test (value=0.010) (**Figure 8B**), a statistical test that gives early events a higher weight in the statistical calculations.

## Discussion

In this study, we characterized the mRNA levels of 19 immune checkpoints associated genes by nCounter in a molecularly characterized series of 80 Brazilian medulloblastomas. We found the absence or shallow mRNA levels of most ICs evaluated, including the currently clinically actionable targets *PDCD1*, *CD274*, and *CTLA4*. At variance, we found higher mRNA levels of *CD24*, *CD47*, *CD276*, and *PVR* genes, and when compared with non-tumor tissue, CD24 and CD276 were significantly overexpressed in the medulloblastomas. These results were further validated *in silico*, showing the association of high *CD276* expression with a worse outcome of medulloblastoma patients from the WNT and Group 4 molecular subgroups, and high levels of *CD24* associated with worse survival in Group 3.

The response to currently actionable immune checkpoint blockade depends on different factors such as immunogenicity and the target’s expression levels. It has been reported that medulloblastoma has a low mutational burden and, therefore, is less immunogenic than other types of tumors, which makes it challenging to target with conventional immunotherapies (37–39). One mechanism associated with a high mutation burden is the presence of MSI (40). The MSI analysis of a subset of our series showed the absence of MSI. These results align with previous studies that reported the absence or very low frequency of MSI in medulloblastomas (41, 42). These characteristics may modulate the tumor’s poor response to PD-1, PD-L1, and CTLA4 blockade (43).

In the present study, we also found the absence or very low mRNA levels of *PDCD1*, *CD274*, and *CTLA4* in both Brazilian and Cavalli cohorts. Moreover, following the mRNA levels, immunohistochemistry for PD-L1 showed no protein expression. Our results are in accordance with other studies that evaluated PD-1 and PD-L1-related gene expression in medulloblastoma (44). Consistent with our findings, Hwang and coauthors reported the absence of PD-L1 expression in 28 medulloblastomas analyzed (45). Moreover, Vermeulen et al. associated the low *PDCD1* levels in the tumor with low lymphocytic infiltration (46). Overall, these findings lower the expectations of initial immune checkpoint inhibitors (ICI) such as pembrolizumab and nivolumab (11, 12). Additionally, enrolling medulloblastoma patients in clinical trials for the abovementioned ICIs lacked appropriate case selection or population size (9). Nevertheless, clinical trials, such as NCT02359565 and NCT03173950, which target PD-1 with pembrolizumab and nivolumab, are recruiting patients with CNS tumors, including medulloblastoma, with a high mutational burden (e.g., rare constitutional MMR-deficiency syndrome – CMMRD) and recurrent or refractory tumors (47).

The combination of different immunotherapy agents is another possible approach for CNS tumors, such as nivolumab and ipilimumab, in treating high-grade pediatric CNS (The NCT03130959 clinical trial). The trial reported delayed disease progression in patients with combined therapy compared to nivolumab monotherapy submitted patients. However, based on our results, the levels of both targets are very low, suggesting limited results of these approaches for the vast majority of medulloblastomas.

The refractory response to PD-1/PD-L1 and CTLA4 blockade by immunologically “cold” tumors like CNS, pancreatic, or prostate cancer gave urgency to finding alternatives to these targets that are mainly dependent on an active immune response (48). Immune checkpoints like LAG-3, TIM-3, PVR, B7-H3, CD47, and IDO1 have been gaining attention as possible substitutes for the initial targets (15). Therefore, we performed the profile of the 19 most preeminent immune checkpoints in our study. We found high mRNA levels in four immune checkpoints related genes, namely *CD24*, *CD276* (B7-H3), *CD47*, and *PVR*, and when compared with non-tumor tissue, we confirmed the selective higher tumor expression of *CD24* and *CD276*. Notably, there are two ongoing clinical trials targeting B7-H3. The NCT04167618 uses an anti-B7-H3 monoclonal antibody (omburtamab) in combination with radiotherapy, and the NCT04743661 uses omburtamab combined with radiotherapy, chemotherapeutic agents like irinotecan or temozolomide and bevacizumab (anti-VEGF). However, none of these clinical trials established the levels of B7-H3 as eligibility criteria nor focused on the molecular subgroup. The function of *B7-H3* was first thought to be immuno-stimulatory, but further associated with several immuno-inhibitory and tumor progressing functions, mainly by its effects on lymphocytic cells, invasion, and angiogenesis (49). Our results are similar to studies that measured the levels of *CD276* expression in different brain tumors, including in medulloblastoma samples and cell lines (50, 51). The analysis performed on the Cavalli et al. microarray dataset and additional dataset comprising 1350 medulloblastomas and 291 normal brain samples corroborate the high mRNA levels of *CD276* across all subgroups and a significantly higher expression than in normal brain tissue. Based on these results, *CD276* (B7-H3) is a strong candidate for future blockade approaches in medulloblastoma.

The higher expressed immune checkpoint-related gene identified in the Brazilian and the Cavalli series was the *CD24.* Similar results were reported by Robson et al. showing high mRNA and protein expression in all medulloblastoma subgroups except for WNT (52). The *CD24* expression profile in the Cavalli et al. cohort is similar to our Brazilian series, also displaying lower mRNA levels in the WNT molecular subgroups. We further observed in our cohort and in a large dataset of medulloblastomas and non-tumor brain tissue that CD24 expression is significantly higher in all molecular medulloblastoma subgroups except WNT, which showed similar expression levels. Additionally, high *CD24* expression was associated with worse survival in the early years in Group 3. The *CD24* primary function is to inhibit macrophage phagocytic capacity by binding Siglec-10 (28). Additionally, *CD24* was suggested to be a tumor-initiating cell marker and has been described with additional functions as an adhesion molecule important for invasion and migration, as well as being capable of activating signaling pathways involved in tumor cell proliferation (52–54). Regarding the tumor immune microenvironment, Bockmayr et al. reported that WNT, SHH, and a subset of Group 4 medulloblastomas present higher myeloid cells infiltration in the tumor and identified an immunosuppressive environment in Group 3 and a subset of Group 4 medulloblastomas as mediated by anti-inflammatory cytokines and immune checkpoints such as TGFβ1, PD-L1, and CTLA4, yet, PD-L1 was absent from their sample analysis (55). Given the higher myeloid infiltration in the SHH and in a subset of Group 4 subgroups, the blockade of CD24 may be a promising approach to induce a more robust anti-tumoral response. Regardless, the CD24 role in medulloblastoma should be further investigated as this may be a promising target for the blockade approach in this tumor.

Another overexpressed immune checkpoint was *CD47*, inhibiting macrophage phagocytic capacity by binding SIRP-α (28, 56). In both Brazilian and *in silico* datasets, the WNT subgroup had significantly higher mRNA levels of *CD47* compared to the other medulloblastoma molecular subgroups. Of note, *CD47* was also highly expressed in non-tumor brain tissue. The *CD47* biological impact in medulloblastoma was investigated by Gholamin et al., that reported high expression levels of *CD47* in medulloblastoma datasets and further showed in mice models that targeting CD47 by the monoclonal antibody magrolimab resulted in enhanced phagocytic capacity and tackled tumor primary and metastatic capacity in a model of Group 3 medulloblastoma (57).

The *PVR* (poliovirus receptor) is a stimulatory and inhibitory immune checkpoint when binding to the costimulatory receptor DNAM-1 and inhibitory receptors TIGIT and CD96, respectively, in immune cells (58). This features grants *PVR* with an immune regulatory capacity that, in the context of the tumor microenvironment, is often switched to a more anti-inflammatory and pro-tumoral activity (58). In our study, *PVR* expression exhibited distinct levels among molecular subgroups and non-tumor brain tissue. Therefore, additional studies are warranted to clarify the PVR role in medulloblastoma’s biology and therapy.

We further assessed the association between immune checkpoint expression levels and patients’ overall survival for the four expressed genes. We could observe statistical differences in the Cavalli dataset, which showed an overall worst survival probability in WNT and Group 4 patients with higher *CD276* mRNA levels. Similar findings were reported in other solid cancer types like non-small cell lung or pancreatic cancer (59). Moreover, high *CD24* mRNA levels were associated with poor survival in the first years following diagnosis in Group 3 medulloblastoma patients.

Altogether, *CD276* and *CD24* are the most attractive immune checkpoints to be targeted in medulloblastoma, given their higher expression in tumors than in non-tumor tissue and their association with worse prognosis. Notably, a nCounter approach for simultaneous and accurate analysis of several immune checkpoint levels can constitute a putative companion diagnostic test for patient immunotherapy selection. Nevertheless, further pre-clinical and clinical studies are needed to confirm these targets’ biological significance and therapeutic impact. One future issue to be addressed is the well-known poor permeability across the blood-brain barrier (BBB), which constitutes a major challenge in advancing medulloblastoma systemic therapy, including immunotherapy (60). In the previously mentioned omburtamab trials (NCT04167618 and NCT04743661), the drug was administered intraventricularly, overcoming, in this way, the BBB obstacle.

Ultimately, we successfully evaluated relevant immune checkpoint targets in the routine FFPE medulloblastoma biopsies. Our study corroborated the lower mRNA levels of the current targeted immune checkpoints, PD-1, PD-L1, and CTLA4. Notably, the immune checkpoints genes coding for CD24 and B7-H3 were highly expressed in medulloblastoma, suggesting that they can constitute more suitable targets for an immune-targeted approach.

## Data availability statement

The all panel expression data is deposited in the “Gene Expression Omnibus” under the accession number GSE223606. It can be found in the following link https://0-www-ncbi-nlm-nih-gov.brum.beds.ac.uk/geo/query/acc.cgi?acc=GSE223606.

## Author contributions

RM and DM; Design of study, organized the database, performed the statistical analysis, results discussion, and drafted the manuscript. LS and LL; Design of study, data generation, results generation, and manuscript review. FP; Data Generation, results discussion, and manuscript review. IS, GT, FS, and LN: Pathological review of tumor samples, data generation results discussion, and manuscript review. CJ and BM: Medical reports analysis, results discussion, and manuscript review. RR: Conception, project coordinator, results discussion, manuscript writing, and review. All authors contributed to the article and approved the submitted version.

## Supplementary material

Supplementary Figure 1 Graphic representation of HAVCR2, LAG3, and TIGIT mRNA normalized expression levels of the 80 medulloblastomas analyzed by nCounter. **(A)** The plot of mRNA levels of all medulloblastomas for HAVCR2, LAG3, and TIGIT **(B)** Plot of HAVCR2, LAG3, and TIGIT mRNA levels by molecular subgroups. The continuous line in each graph marks the background threshold of 20 mRNA normalized expression counts. The plots were obtained through GraphPad Prism 8.
