# Supplementary figures and images for: Digital expression profile of immune checkpoint genes in medulloblastomas identifies CD24 and CD276 as putative immunotherapy targets
Source: Front Immunol. 2023 Feb 7;14:1062856. doi: 10.3389/fimmu.2023.1062856 (PMC9941636; doi:10.3389/fimmu.2023.1062856)

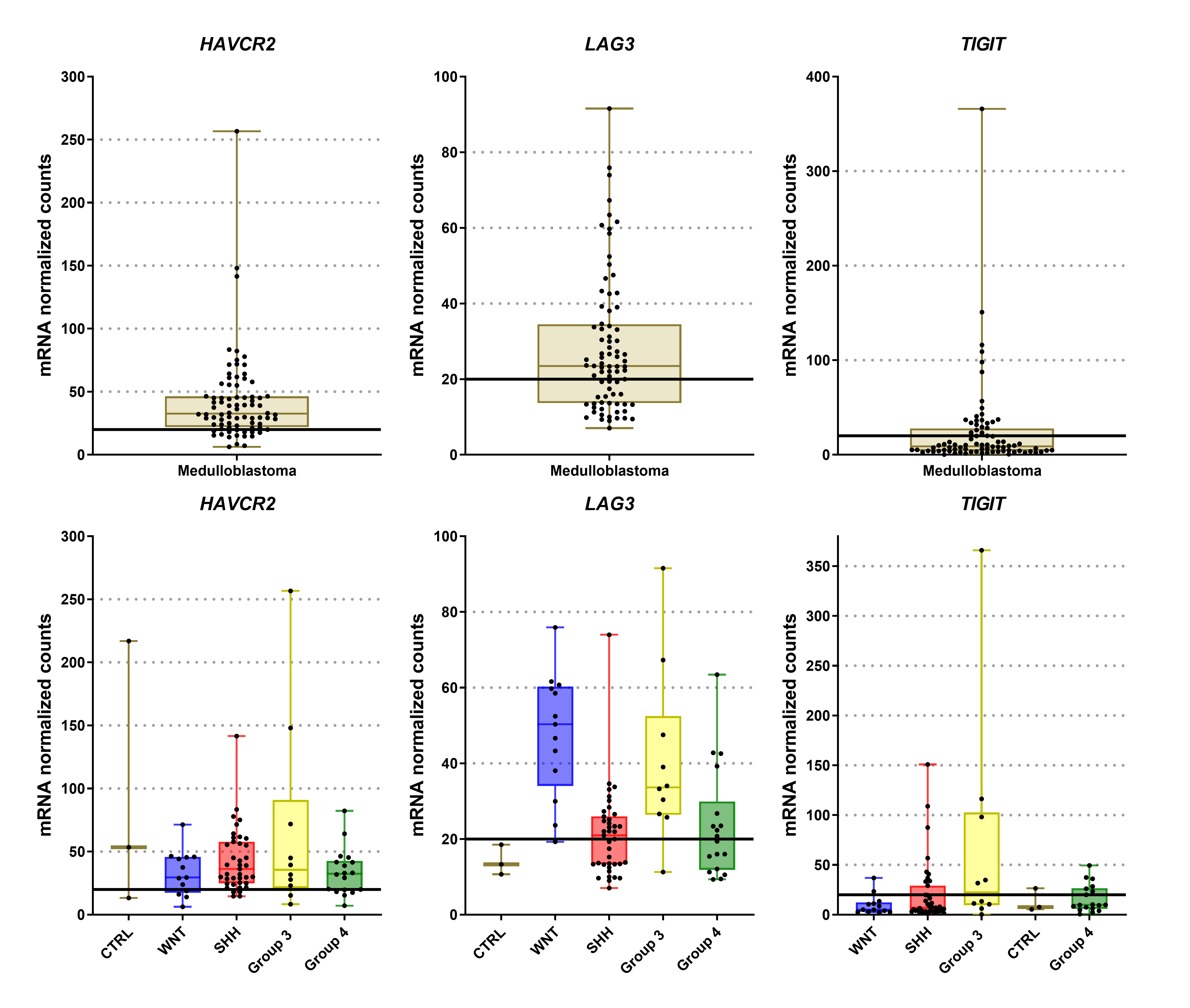

Supplement: Supplementary Figure 1 — Graphic representation of HAVCR2, LAG3, and TIGIT mRNA normalized expression levels of the 80 medulloblastomas analyzed by nCounter. (A) The plot of mRNA levels of all medulloblastomas for HAVCR2, LAG3, and TIGIT (B) Plot of HAVCR2, LAG3, and TIGIT mRNA levels by molecular subgroups. The continuous line in each graph marks the background threshold of 20 mRNA normalized expression counts. The plots were obtained through GraphPad Prism 8. [file Image_1.tif]
