# Supplementary material for: Digital expression profile of immune checkpoint genes in medulloblastomas identifies CD24 and CD276 as putative immunotherapy targets
Source: Front Immunol. 2023 Feb 7;14:1062856. doi: 10.3389/fimmu.2023.1062856 (PMC9941636; doi:10.3389/fimmu.2023.1062856)
Supplement: Supplementary file 2 [file Table_1.docx]

**Supplementary table 1:** Descriptive statistics of the evaluated immune checkpoints in each subgroup of the Brazilian cohort. This description contains the mean, standard deviation, minimum and maximum expression values of each gene.

| Brazilian dataset | | | |  | |  | |  | | |  | | |  | |  | |  | |  | | |  | | |  | |  | |  | | |  | |  | | |  | |  | |  |  |
| --- | --- | --- | --- | --- | --- | --- | --- | --- | --- | --- | --- | --- | --- | --- | --- | --- | --- | --- | --- | --- | --- | --- | --- | --- | --- | --- | --- | --- | --- | --- | --- | --- | --- | --- | --- | --- | --- | --- | --- | --- | --- | --- | --- |
|  | **WNT (n = 13)** | | | | | | | |  | | | **SHH (n = 39)** | | | | | | | | |  | | | **Group 3 (n = 10)** | | | | | | | | | |  | | **Group 4 (n = 18)** | | | | | | | |
| **Genes** | **Mean** | **S.D.** | **Min.** | | **Max.** | |  | | | **Mean** | | | **S.D.** | | **Min.** | | **Max.** | |  | | | **Mean** | | | **S.D.** | | **Min.** | | **Max.** | |  | **Mean** | | | | | **S.D.** | | **Min.** | | **Max.** | | |
| ***BTLA*** | **3.9** | 1.6 | 1.1 | | 6.6 | |  | | | **15.9** | | | 21.1 | | 0.6 | | 99.8 | |  | | | **20.6** | | | 16.0 | | 2.5 | | 42.8 | |  | **12.8** | | | | | 12.8 | | 1.2 | | 43.0 | | |
| ***CD160*** | **2.8** | 1.4 | 0.9 | | 5.6 | |  | | | **17.7** | | | 23.8 | | 0.8 | | 96.2 | |  | | | **28.3** | | | 30.6 | | 0.4 | | 94.8 | |  | **13.8** | | | | | 14.8 | | 0.5 | | 47.2 | | |
| ***CD24*** | **763.7** | 275.2 | 76.2 | | 1112.6 | |  | | | **1889.1** | | | 648.3 | | 720.6 | | 3384.6 | |  | | | **1459.4** | | | 516.1 | | 800.4 | | 2443.4 | |  | **1710.4** | | | | | 707.8 | | 89.4 | | 3122.2 | | |
| ***CD244*** | **2.3** | 1.1 | 0.7 | | 4.6 | |  | | | **2.9** | | | 1.9 | | 0.6 | | 11.6 | |  | | | **7.9** | | | 11.2 | | 0.6 | | 38.0 | |  | **2.9** | | | | | 2.1 | | 0.8 | | 9.3 | | |
| ***CD274*** | **5.8** | 2.9 | 2.2 | | 13.4 | |  | | | **10.1** | | | 12.2 | | 1.1 | | 61.4 | |  | | | **8.7** | | | 7.5 | | 1.1 | | 24.2 | |  | **7.8** | | | | | 9.1 | | 0.8 | | 32.0 | | |
| ***CD276*** | **633.4** | 143.4 | 431.6 | | 971.6 | |  | | | **432.0** | | | 98.1 | | 220.9 | | 619.6 | |  | | | **476.2** | | | 127.1 | | 299.0 | | 673.6 | |  | **487.3** | | | | | 93.5 | | 323.6 | | 709.5 | | |
| ***CD47*** | **772.6** | 220.3 | 508.6 | | 1400.8 | |  | | | **434.2** | | | 199.0 | | 126.7 | | 929.3 | |  | | | **211.7** | | | 139.2 | | 67.3 | | 508.4 | |  | **305.9** | | | | | 138.8 | | 98.7 | | 599.3 | | |
| ***CD48*** | **9.5** | 6.9 | 1.5 | | 26.7 | |  | | | **14.8** | | | 17.5 | | 0.6 | | 93.7 | |  | | | **16.5** | | | 15.5 | | 1.5 | | 55.6 | |  | **10.1** | | | | | 8.8 | | 2.1 | | 33.7 | | |
| ***CD80*** | **3.5** | 1.7 | 0.5 | | 6.4 | |  | | | **13.7** | | | 18.3 | | 0.6 | | 78.3 | |  | | | **13.1** | | | 12.4 | | 0.8 | | 32.7 | |  | **9.2** | | | | | 10.0 | | 0.8 | | 33.7 | | |
| ***CD86*** | **7.1** | 4.2 | 2.0 | | 16.0 | |  | | | **16.4** | | | 16.4 | | 1.6 | | 69.1 | |  | | | **16.3** | | | 11.4 | | 2.6 | | 33.3 | |  | **10.6** | | | | | 8.4 | | 1.5 | | 30.3 | | |
| ***CEACAM1*** | **3.4** | 2.1 | 1.1 | | 6.6 | |  | | | **12.7** | | | 16.8 | | 0.8 | | 79.8 | |  | | | **14.1** | | | 13.5 | | 0.9 | | 42.8 | |  | **7.3** | | | | | 6.5 | | 0.5 | | 26.4 | | |
| ***CTLA4*** | **4.2** | 2.1 | 1.3 | | 9.0 | |  | | | **14.6** | | | 15.0 | | 0.6 | | 69.6 | |  | | | **17.2** | | | 15.5 | | 1.5 | | 42.8 | |  | **8.5** | | | | | 7.0 | | 1.3 | | 27.8 | | |
| ***HAVCR2*** | **33.0** | 17.1 | 6.3 | | 71.4 | |  | | | **43.0** | | | 24.6 | | 14.7 | | 141.5 | |  | | | **66.7** | | | 73.9 | | 8.3 | | 256.6 | |  | **33.7** | | | | | 18.0 | | 7.1 | | 82.3 | | |
| ***IDO1*** | **8.3** | 5.1 | 1.3 | | 17.8 | |  | | | **16.9** | | | 16.7 | | 1.6 | | 73.7 | |  | | | **18.0** | | | 15.2 | | 0.4 | | 52.3 | |  | **12.8** | | | | | 9.2 | | 1.7 | | 30.3 | | |
| ***LAG3*** | **47.7** | 15.9 | 19.3 | | 75.9 | |  | | | **21.1** | | | 11.5 | | 7.0 | | 74.0 | |  | | | **40.7** | | | 22.0 | | 11.3 | | 91.6 | |  | **23.6** | | | | | 14.2 | | 9.3 | | 63.4 | | |
| ***PDCD1*** | **3.2** | 2.3 | 0.5 | | 7.7 | |  | | | **13.5** | | | 17.2 | | 0.8 | | 75.2 | |  | | | **12.5** | | | 14.6 | | 0.9 | | 42.5 | |  | **10.1** | | | | | 13.6 | | 0.5 | | 45.5 | | |
| ***PVR*** | **187.4** | 67.9 | 103.7 | | 345.7 | |  | | | **86.2** | | | 34.8 | | 12.7 | | 170.1 | |  | | | **124.9** | | | 75.8 | | 15.2 | | 251.9 | |  | **50.2** | | | | | 32.4 | | 6.4 | | 110.2 | | |
| ***TIGIT*** | **9.9** | 9.7 | 2.3 | | 36.9 | |  | | | **20.8** | | | 31.4 | | 1.5 | | 150.8 | |  | | | **68.9** | | | 106.0 | | 0.2 | | 366.0 | |  | **16.3** | | | | | 13.7 | | 0.6 | | 49.4 | | |
| ***TNFSF14*** | **3.0** | 1.0 | 1.3 | | 4.7 | |  | | | **6.1** | | | 7.7 | | 0.6 | | 44.1 | |  | | | **11.0** | | | 13.8 | | 0.8 | | 47.5 | |  | **6.0** | | | | | 5.9 | | 0.5 | | 22.5 | | |
| Abbreviations: S.D. = Standard Deviation; Min. = Minimum; Max. = Maximum; | | | | | | | | | | | | | | | | | | | | | | | | | | | | | | | | | | | | | | | | | | |  |
| "n" stands for sample size | | | | | | | | | | | | | | | | | | | | | | | | | | | | | | | | | | | | | | | | | | |  |

**Supplementary table 2:** Descriptive statistics of the evaluated immune checkpoints in each subgroup of the Cavalli et al 763 medulloblastomas dataset. This description contains the mean, standard deviation, minimum and maximum values of each gene.

| Cavalli dataset | |  |  |  |  |  |  |  |  |  |  |  |  |  |  | |  | |  |  |  |  |
| --- | --- | --- | --- | --- | --- | --- | --- | --- | --- | --- | --- | --- | --- | --- | --- | --- | --- | --- | --- | --- | --- | --- |
|  | **WNT (n = 70)** | | | |  | **SHH (n = 223)** | | | |  | **Group 3 (n = 144)** | | | | |  | | **Group 4 (n = 326)** | | | | |
| **Genes** | **Mean** | **S.D.** | **Min.** | **Max.** |  | **Mean** | **S.D.** | **Min.** | **Max.** |  | **Mean** | **S.D.** | **Min.** | **Max.** |  | | **Mean** | | **S.D.** | **Min.** | **Max.** |  |
| ***BTLA*** | **15.4** | 2.2 | 9.8 | 21.3 |  | **15.8** | 3.1 | 10.0 | 35.3 |  | **16.8** | 3.7 | 9.0 | 35.9 |  | | **15.6** | | 2.4 | 10.4 | 24.7 |  |
| ***CD160*** | **9.1** | 1.0 | 7.3 | 13.3 |  | **9.4** | 2.7 | 6.6 | 35.2 |  | **9.6** | 1.4 | 7.1 | 14.3 |  | | **9.1** | | 1.2 | 6.9 | 14.9 |  |
| ***CD24*** | **554.2** | 285.5 | 15.6 | 1443.6 |  | **1086.9** | 337.3 | 12.6 | 2332.1 |  | **823.4** | 364.8 | 16.1 | 1967.2 |  | | **1276.7** | | 350.2 | 418.9 | 2293.8 |  |
| ***CD244*** | **12.1** | 1.7 | 9.1 | 17.2 |  | **12.1** | 1.6 | 9.0 | 17.9 |  | **12.5** | 2.2 | 8.3 | 24.0 |  | | **11.8** | | 1.7 | 8.3 | 18.4 |  |
| ***CD274*** | **21.3** | 11.0 | 10.6 | 76.5 |  | **16.3** | 8.6 | 9.9 | 77.4 |  | **14.7** | 5.4 | 8.8 | 62.2 |  | | **13.1** | | 2.8 | 9.3 | 39.3 |  |
| ***CD276*** | **424.6** | 90.1 | 213.2 | 586.9 |  | **297.0** | 82.5 | 122.2 | 594.4 |  | **315.0** | 87.6 | 150.6 | 592.0 |  | | **377.8** | | 78.1 | 170.6 | 728.6 |  |
| ***CD47*** | **1224.5** | 289.8 | 723.1 | 2113.4 |  | **1011.6** | 359.0 | 177.9 | 3273.0 |  | **646.2** | 313.4 | 128.4 | 1685.1 |  | | **727.5** | | 289.9 | 212.2 | 1674.3 |  |
| ***CD48*** | **32.8** | 23.3 | 17.0 | 204.2 |  | **32.7** | 10.6 | 18.1 | 95.4 |  | **27.0** | 7.8 | 16.7 | 70.4 |  | | **29.3** | | 10.2 | 15.6 | 97.0 |  |
| ***CD80*** | **17.6** | 4.9 | 12.6 | 37.0 |  | **15.7** | 3.1 | 11.3 | 29.8 |  | **17.0** | 10.4 | 11.7 | 135.4 |  | | **15.1** | | 2.7 | 10.5 | 29.7 |  |
| ***CD86*** | **30.0** | 13.8 | 15.4 | 93.4 |  | **36.4** | 15.0 | 14.8 | 104.1 |  | **27.1** | 11.9 | 14.4 | 108.6 |  | | **29.5** | | 9.9 | 14.5 | 82.0 |  |
| ***CEACAM1*** | **17.2** | 2.5 | 12.7 | 24.7 |  | **19.7** | 6.7 | 12.6 | 88.3 |  | **17.3** | 5.0 | 11.7 | 61.9 |  | | **17.2** | | 2.8 | 11.4 | 31.7 |  |
| ***CTLA4*** | **12.9** | 1.9 | 10.2 | 19.8 |  | **13.9** | 2.2 | 10.4 | 23.1 |  | **13.7** | 2.0 | 10.8 | 22.2 |  | | **13.3** | | 2.0 | 9.3 | 21.5 |  |
| ***HAVCR2*** | **52.3** | 23.0 | 28.2 | 154.4 |  | **62.9** | 26.0 | 29.8 | 271.4 |  | **44.9** | 16.4 | 25.7 | 147.2 |  | | **47.9** | | 15.4 | 25.3 | 159.0 |  |
| ***IDO1*** | **15.1** | 3.5 | 10.6 | 31.9 |  | **15.1** | 3.8 | 10.7 | 41.7 |  | **25.9** | 132.8 | 10.6 | 1612.1 |  | | **13.9** | | 1.9 | 9.8 | 21.7 |  |
| ***LAG3*** | **69.9** | 15.1 | 40.4 | 127.0 |  | **57.0** | 16.5 | 29.6 | 223.2 |  | **67.0** | 16.8 | 35.7 | 127.0 |  | | **61.1** | | 14.1 | 31.0 | 125.3 |  |
| ***PDCD1*** | **45.3** | 7.4 | 29.5 | 68.9 |  | **46.4** | 7.7 | 31.3 | 80.2 |  | **46.8** | 7.6 | 33.5 | 81.0 |  | | **49.9** | | 7.9 | 33.7 | 77.3 |  |
| ***PVR*** | **252.0** | 68.4 | 66.2 | 482.6 |  | **150.8** | 41.5 | 67.2 | 374.2 |  | **163.6** | 67.4 | 33.7 | 365.3 |  | | **84.0** | | 44.5 | 25.0 | 266.1 |  |
| ***TIGIT*** | **12.7** | 2.0 | 9.3 | 19.5 |  | **13.2** | 2.7 | 8.3 | 27.0 |  | **12.2** | 3.3 | 8.7 | 41.8 |  | | **12.3** | | 2.0 | 8.5 | 27.1 |  |
| ***TNFSF14*** | **45.0** | 9.1 | 30.6 | 75.5 |  | **40.0** | 6.2 | 27.6 | 66.7 |  | **43.1** | 8.3 | 30.3 | 77.9 |  | | **41.1** | | 6.3 | 27.0 | 65.6 |  |
| Abbreviations: S.D. = Standard Deviation; Min. = Minimum; Max. = Maximum; | | | | | | | | | | | | | | | | | | | | | | |
| "n" stands for sample size | | | | | | | | | | | | | | | | | | | | | | |

**Supplementary table 3:** Statistical values for the comparisons of immune checkpoint mRNA expression between molecular subgroups from the 80 medulloblastomas cohort analyzed through nCounter. The statistical values were obtained through One-Way ANOVA. p-value reporting symbols: * <0.05, ** < 0.01, *** < 0.001 and ns > 0.05 if no significance is observed.

| **ANOVA/Welch** | |
| --- | --- |
|  | **Sig.** |
| BTLA | ns |
| CD160 | ns |
| **CD24** | ******* |
| CD244 | ns |
| CD274 | ns |
| **CD276** | ******* |
| **CD47** | ******* |
| CD48 | ns |
| CD80 | ns |
| CD86 | * |
| CEACAM1 | ns |
| CTLA4 | ns |
| HAVCR2 | ns |
| IDO1 | ns |
| LAG3 | *** |
| PDCD1 | ns |
| **PVR** | ******* |
| TIGIT | ns |
| TNFSF14 | ns |

**Supplementary table 4:** Multiple comparison analysis through Tukey HSD method for CD24, CD276, CD47 and PVR mRNA levels of 80 medulloblastomas included in the 80 medulloblastomas cohort analyzed through nCounter. p-value reporting symbols: * <0.05, ** < 0.01, *** < 0.001 and ns > 0.05 if no significance is observed.

| **Tukey HSD** | | | | | |
| --- | --- | --- | --- | --- | --- |
|  |  | **SHH** | **G3** | **G4** | **CTRL** |
| **CD24** | **WNT** | *** | ns | ** | ns |
|  | **SHH** |  | ns | ns | *** |
|  | **G3** |  |  | ns | * |
|  | **G4** |  |  |  | ** |
| **CD276** | **WNT** | *** | * | ** | *** |
|  | **SHH** |  | ns | ns | ** |
|  | **G3** |  |  | ns | ** |
|  | **G4** |  |  |  | ** |
| **CD47** | **WNT** | *** | *** | *** | ns |
|  | **SHH** |  | * | ns | ns |
|  | **G3** |  |  | ns | ** |
|  | **G4** |  |  |  | ** |
| **PVR** | **WNT** | *** | * | *** | ns |
|  | **SHH** |  | ns | ns | ns |
|  | **G3** |  |  | ** | ns |
|  | **G4** |  |  |  | ** |

**Supplementary table 5:** Statistical values for the comparisons of CD24, CD276, CD47 and PVR mRNA relative expression between molecular subgroups from the medulloblastoma Batch data cohort. The statistical values were obtained through One-Way ANOVA. None of the evaluated genes showed homogeneity of variances, therefore it was applied the Welch test for robust comparisons. p-value reporting symbols: * <0.05, ** < 0.01, *** < 0.001 and ns > 0.05 if no significance is observed.

| **Welch** | |
| --- | --- |
|  | **Sig.** |
| CD24 | ******* |
| CD276 | ******* |
| CD47 | ******* |
| PVR | ******* |

**Supplementary table 6:** Multiple comparison analysis through Games-Howell method for CD24, CD47, CD276 and PVR mRNA levels of 80 medulloblastomas included in the medulloblastoma batch data cohort. p-value reporting symbols: * <0.05, ** < 0.01, *** < 0.001 and ns > 0.05 if no significance is observed.

| **Games-Howell** | | | | | |
| --- | --- | --- | --- | --- | --- |
|  |  | **SHH** | **G3** | **G4** | **Normal** |
| **CD24** | **WNT** | *** | ** | *** | ns |
|  | **SHH** |  | *** | ns | *** |
|  | **G3** |  |  | *** | *** |
|  | **G4** |  |  |  | *** |
| **CD276** | **WNT** | *** | *** | *** | *** |
|  | **SHH** |  | ** | *** | *** |
|  | **G3** |  |  | *** | *** |
|  | **G4** |  |  |  | *** |
| **CD47** | **WNT** | *** | *** | *** | *** |
|  | **SHH** |  | *** | *** | ns |
|  | **G3** |  |  | *** | *** |
|  | **G4** |  |  |  | *** |
| **PVR** | **WNT** | *** | *** | *** | *** |
|  | **SHH** |  | ns | *** | * |
|  | **G3** |  |  | *** | ** |
|  | **G4** |  |  |  | *** |

**Supplementary table 7:** Survival analysis of our 80 medulloblastoma cases made by the Log-rank and Gehan-Breslow-Wilcoxon (GBW) methods. The table shows the sample size of each molecular subgroup (N), the median overall survival (Median OS) and the p-value for the Log-rank and GBW tests (Log-rank/GBW).

|  |  | **WNT** | | |  | **SHH** | | |  | **Group 3** | | |  | **Group 4** | | |
| --- | --- | --- | --- | --- | --- | --- | --- | --- | --- | --- | --- | --- | --- | --- | --- | --- |
|  |  | **N** | **Median OS** | **Log-rank/GBW** |  | **N** | **Median OS** | **Log-rank/GBW** |  | **N** | **Median OS** | **Log-rank/GBW** |  | **N** | **Median OS** | **Log-rank/GBW** |
| ***BTLA*** | High | 5 | Undefined | 0.85/0.39 |  | 19 | 74.08 | 0.76/0.99 |  | 5 | 36.53 | 0.26/0.29 |  | 9 | 42.67 | 0.26/0.32 |
|  | Low | 7 | Undefined |  |  | 19 | 83.97 |  |  | 5 | Undefined |  |  | 9 | Undefined |  |
| ***CD160*** | High | 6 | 34.71 | 0.31/0.39 |  | 19 | 74.08 | 0.94/0.71 |  | 5 | 36.53 | 0.26/0.29 |  | 9 | Undefined | 0.94/0.88 |
|  | Low | 6 | Undefined |  |  | 19 | 83.97 |  |  | 5 | Undefined |  |  | 9 | Undefined |  |
| ***CD24*** | High | 5 | Undefined | 0.54/0.43 |  | 19 | 335 | 0.28/0.54 |  | 5 | Undefined | 0.99/> 0.99 |  | 9 | Undefined | 0.76/0.88 |
|  | Low | 7 | Undefined |  |  | 19 | 74.08 |  |  | 5 | Undefined |  |  | 9 | Undefined |  |
| ***CD244*** | High | 6 | Undefined | 0.18/0.15 |  | 20 | 24.05 | 0.22/0.059 |  | 5 | Undefined | 0.99/> 0.99 |  | 9 | 42.67 | 0.23/0.25 |
|  | Low | 6 | 32.84 |  |  | 18 | 109.8 |  |  | 5 | Undefined |  |  | 9 | Undefined |  |
| ***CD274*** | High | 5 | Undefined | 0.052/0.056 |  | 19 | 74.08 | 0.92/0.88 |  | 5 | 36.53 | 0.26/0.29 |  | 9 | 42.67 | 0.24/0.27 |
|  | Low | 7 | 10.28 |  |  | 19 | 83.97 |  |  | 5 | Undefined |  |  | 9 | Undefined |  |
| ***CD276*** | High | 5 | Undefined | 0.05/0.056 |  | 18 | 83.97 | 0.72/0.80 |  | 5 | 36.53 | 0.26/0.29 |  | 9 | Undefined | 0.80/> 0.99 |
|  | Low | 7 | 10.28 |  |  | 20 | 74.08 |  |  | 5 | Undefined |  |  | 9 | Undefined |  |
| ***CD47*** | High | 6 | Undefined | 0.99/0.99 |  | 19 | 61 | 0.53/0.39 |  | 5 | Undefined | 0.15/0.12 |  | 9 | Undefined | 0.52/0.40 |
|  | Low | 6 | Undefined |  |  | 19 | 109.8 |  |  | 5 | 12.32 |  |  | 9 | Undefined |  |
| ***CD48*** | High | 6 | Undefined | 0.84**/**0.70 |  | 19 | 74.08 | 0.90/0.88 |  | 5 | 36.53 | 0.26/0.29 |  | 9 | 42.67 | 0.24/0.27 |
|  | Low | 6 | Undefined |  |  | 19 | 109.8 |  |  | 5 | Undefined |  |  | 9 | Undefined |  |
| ***CD80*** | High | 6 | 50.84 | 0.31/0.39 |  | 19 | 74.08 | 0.74/0.67 |  | 5 | 36.53 | 0.26/0.29 |  | 9 | Undefined | 0.94/0.88 |
|  | Low | 6 | Undefined |  |  | 19 | 109.8 |  |  | 5 | Undefined |  |  | 9 | Undefined |  |
| ***CD86*** | High | 6 | Undefined | 0.84/0.70 |  | 19 | 74.08 | 0.87/0.73 |  | 5 | 36.53 | 0.26/0.29 |  | 9 | Undefined | 0.71/0.64 |
|  | Low | 6 | Undefined |  |  | 19 | Undefined |  |  | 5 | Undefined |  |  | 9 | Undefined |  |
| ***CEACAM1*** | High | 6 | Undefined | 0.84/0.70 |  | 19 | 74.08 | 0.63/0.46 |  | 5 | Undefined | 0.90/0.81 |  | 9 | Undefined | 0.49/0.50 |
|  | Low | 6 | Undefined |  |  | 19 | 109.8 |  |  | 5 | Undefined |  |  | 9 | 42.67 |  |
| ***CTLA4*** | High | 6 | Undefined | 0.18/0.15 |  | 19 | 130.4 | 0.35/0.29 |  | 5 | 36.53 | 0.26/0.29 |  | 9 | 42.67 | 0.26/0.32 |
|  | Low | 6 | 32.84 |  |  | 19 | 83.97 |  |  | 5 | Undefined |  |  | 9 | Undefined |  |
| ***HAVCR2*** | High | 6 | Undefined | 0.84/0.70 |  | 19 | 130.4 | 0.12/0.12 |  | 5 | 36.53 | 0.26/0.29 |  | 9 | Undefined | 0.94**/**0.88 |
|  | Low | 6 | Undefined |  |  | 19 | 31.08 |  |  | 5 | Undefined |  |  | 9 | Undefined |  |
| ***IDO1*** | High | 6 | 6.11 | **0.018/0.021** |  | 19 | 74.08 | 0.87/0.52 |  | 5 | 36.53 | 0.26/0.29 |  | 9 | 42.67 | 0.26/0.32 |
|  | Low | 6 | Undefined |  |  | 19 | 83.97 |  |  | 5 | Undefined |  |  | 9 | Undefined |  |
| ***LAG3*** | High | 5 | 10.28 | 0.15/0.21 |  | 19 | 83.97 | 0.70/0.31 |  | 5 | Undefined | 0.80/0.64 |  | 9 | Undefined | 0.33/0.22 |
|  | Low | 7 | Undefined |  |  | 19 | 109.8 |  |  | 5 | Undefined |  |  | 9 | Undefined |  |
| ***PDCD1*** | High | 6 | 50.84 | 0.31/0.39 |  | 19 | 61 | 0.72/0.65 |  | 5 | 36.53 | 0.26/0.29 |  | 9 | 42.67 | 0.28/0.41 |
|  | Low | 6 | Undefined |  |  | 19 | 109.8 |  |  | 5 | Undefined |  |  | 9 | Undefined |  |
| ***PVR*** | High | 6 | Undefined | 0.23/0.22 |  | 19 | 335 | 0.07/0.15 |  | 5 | Undefined | 0.90/0.81 |  | 9 | 42.67 | 0.16/0.19 |
|  | Low | 6 | 50.84 |  |  | 19 | 61 |  |  | 5 | Undefined |  |  | 9 | Undefined |  |
| ***TIGIT*** | High | 6 | Undefined | 0.84/0.70 |  | 19 | 74.08 | 0.63/0.89 |  | 5 | 12.32 | 0.15/0.12 |  | 9 | 42.67 | 0.28/0.41 |
|  | Low | 6 | Undefined |  |  | 19 | Undefined |  |  | 5 | Undefined |  |  | 9 | Undefined |  |
| ***TNFSF14*** | High | 5 | Undefined | 0.41/0.39 |  | 19 | 74.08 | 0.74/0.75 |  | 5 | 12.32 | 0.15/0.12 |  | 9 | 42.21 | **0.028/0.037** |
|  | Low | 7 | Undefined |  |  | 19 | 83.97 |  |  | 5 | Undefined |  |  | 9 | Undefined |  |

**Supplementary Table 8:** Survival analysis of the Cavalli et al 763 medulloblastomas made by the Log-rank and Gehan-Breslow-Wilcoxon (GBW) method. The table shows the sample size of each expression group (N), the median overall survival (Median OS) and the p-value for the Log-rank and GBW tests (Log-rank/GBW).

|  |  | **WNT** | | |  | **SHH** | | |  | **Group 3** | | |  | **Group 4** | | |
| --- | --- | --- | --- | --- | --- | --- | --- | --- | --- | --- | --- | --- | --- | --- | --- | --- |
|  |  | **N** | **Median OS** | **Log-rank/GBW** |  | **N** | **Median OS** | **Log-rank/GBW** |  | **N** | **Median OS** | **Log-rank/GBW** |  | **N** | **Median OS** | **Log-rank/GBW** |
| ***BTLA*** | High | 32 | 160 | 0.76/0.53 |  | 87 | Undefined | 0.82/0.99 |  | 60 | 96 | 0.71/0.79 |  | 136 | 168 | 0.84/0.46 |
|  | Low | 31 | Undefined |  |  | 85 | Undefined |  |  | 52 | 179.98 |  |  | 128 | Undefined |  |
| ***CD160*** | High | 32 | 160 | 0.14/0.23 |  | 92 | Undefined | 0.21/0.042 |  | 50 | 124 | 0.18/**0.033** |  | 135 | 135.6 | 0.10/0.18 |
|  | Low | 31 | Undefined |  |  | 80 | Undefined |  |  | 62 | Undefined |  |  | 128 | 264 |  |
| ***CD24*** | High | 32 | Undefined | 0.51/0.25 |  | 88 | Undefined | 0.80/0.38 |  | 53 | 124 | 0.11/**0.010** |  | 134 | 122 | 0.42/0.84 |
|  | Low | 31 | 187 |  |  | 84 | Undefined |  |  | 59 | 80 |  |  | 129 | Undefined |  |
| ***CD244*** | High | 32 | Undefined | **0.028**/0.13 |  | 92 | Undefined | 0.24/0.22 |  | 61 | Undefined | 0.091/0.093 |  | 134 | 136 | 0.30/0.84 |
|  | Low | 31 | 160 |  |  | 80 | Undefined |  |  | 51 | 58 |  |  | 129 | 264 |  |
| ***CD274*** | High | 32 | Undefined | 0.41/0.20 |  | 86 | Undefined | 0.22/0.28 |  | 53 | 180 | 0.58/0.72 |  | 125 | 168 | 0.10/0.11 |
|  | Low | 31 | 166 |  |  | 86 | Undefined |  |  | 59 | 124 |  |  | 138 | 264 |  |
| ***CD276*** | High | 32 | 159 | **0.040**/0.15 |  | 87 | Undefined | 0.78/0.31 |  | 55 | 180 | 0.90/0.87 |  | 129 | 121 | **0.0011/0.0049** |
|  | Low | 31 | Undefined |  |  | 85 | Undefined |  |  | 57 | 124 |  |  | 134 | 264 |  |
| ***CD47*** | High | 32 | Undefined | 0.16/0.21 |  | 88 | Undefined | 0.85/0.81 |  | 60 | 124 | 0.73/0.71 |  | 131 | 168 | 0.63/0.53 |
|  | Low | 31 | Undefined |  |  | 84 | Undefined |  |  | 52 | 180 |  |  | 132 | 136 |  |
| ***CD48*** | High | 32 | Undefined | 0.80/0.44 |  | 89 | Undefined | 0.78/0.41 |  | 60 | 96 | 0.76/0.73 |  | 130 | 136 | 0.50/0.78 |
|  | Low | 31 | Undefined |  |  | 83 | Undefined |  |  | 52 | Undefined |  |  | 133 | 168 |  |
| ***CD80*** | High | 33 | 166 | 0.38/0.19 |  | 88 | Undefined | 0.59/0.44 |  | 56 | 180 | 0.77/0.54 |  | 132 | 168 | 0.37/0.63 |
|  | Low | 30 | Undefined |  |  | 84 | Undefined |  |  | 56 | 96 |  |  | 131 | 264 |  |
| ***CD86*** | High | 32 | Undefined | 0.13/0.19 |  | 85 | Undefined | 0.25/0.64 |  | 57 | 80.9 | 0.26/0.51 |  | 129 | 136 | 0.22/0.59 |
|  | Low | 31 | Undefined |  |  | 87 | Undefined |  |  | 55 | 180 |  |  | 134 | Undefined |  |
| ***CEACAM1*** | High | 32 | 159 | **0.040**/0.15 |  | 90 | Undefined | 0.32/0.27 |  | 56 | Undefined | 0.64/0.78 |  | 139 | 168 | 0.39/0.35 |
|  | Low | 31 | Undefined |  |  | 82 | Undefined |  |  | 56 | 96 |  |  | 124 | Undefined |  |
| ***CTLA4*** | High | 32 | 159 | 0.75/0.53 |  | 82 | Undefined | 0.86/0.75 |  | 60 | 96 | 0.43/0.74 |  | 130 | 168 | 0.26/0.24 |
|  | Low | 31 | Undefined |  |  | 90 | Undefined |  |  | 52 | Undefined |  |  | 133 | Undefined |  |
| ***HAVCR2*** | High | 32 | Undefined | 0.90/0.51 |  | 84 | Undefined | 0.63/0.31 |  | 59 | 124 | 0.45/0.40 |  | 128 | 264 | 0.45/0.13 |
|  | Low | 31 | Undefined |  |  | 88 | Undefined |  |  | 53 | 180 |  |  | 135 | 168 |  |
| ***IDO1*** | High | 32 | Undefined | 0.13/0.17 |  | 81 | Undefined | 0.53/0.97 |  | 57 | Undefined | 0.88/0.58 |  | 136 | Undefined | 0.79/0.61 |
|  | Low | 31 | 159 |  |  | 91 | Undefined |  |  | 55 | 124 |  |  | 127 | 168 |  |
| ***LAG3*** | High | 32 | 159 | 0.79/0.51 |  | 92 | Undefined | 0.37/0.43 |  | 51 | 180 | 0.24/0.20 |  | 129 | 168 | 0.50/0.64 |
|  | Low | 31 | Undefined |  |  | 80 | Undefined |  |  | 61 | 96 |  |  | 134 | 264 |  |
| ***PDCD1*** | High | 32 | Undefined | 0.096/0.16 |  | 92 | Undefined | 0.48/0.45 |  | 54 | 124 | 0.21/0.11 |  | 135 | 168 | 0.61/0.56 |
|  | Low | 31 | 159 |  |  | 80 | Undefined |  |  | 58 | 80 |  |  | 128 | Undefined |  |
| ***PVR*** | High | 32 | 160 | 0.70/0.53 |  | 91 | Undefined | 0.87/0.55 |  | 54 | Undefined | 0.49/0.99 |  | 135 | 168 | 0.72/0.99 |
|  | Low | 31 | Undefined |  |  | 81 | Undefined |  |  | 58 | 80 |  |  | 128 | 136 |  |
| ***TIGIT*** | High | 32 | Undefined | 0.31/0.31 |  | 90 | Undefined | 0.67/0.48 |  | 60 | 180 | 0.38/0.37 |  | 138 | 168 | 0.53/0.38 |
|  | Low | 31 | Undefined |  |  | 82 | Undefined |  |  | 52 | 80 |  |  | 125 | Undefined |  |
| ***TNFSF14*** | High | 32 | Undefined | 0.74/0.42 |  | 88 | Undefined | **0.038**/0.20 |  | 56 | 96 | 0.45/0.76 |  | 132 | 168 | 0.58/0.89 |
|  | Low | 31 | Undefined |  |  | 84 | Undefined |  |  | 56 | 180 |  |  | 131 | 264 |  |
